# Multidimensional transdiagnostic inventory of psychopathology: development and initial validation

**DOI:** 10.3389/fpsyg.2026.1917064

**Published:** 2026-10-28

**Authors:** Mark A. Blais, Hannah Cai, Till J. Adam, Christoph U. Correll

**Affiliations:** 1 Department of Psychiatry, Massachusetts General Hospital, Boston, MA, United States; 2 Harvard Medical School, Boston, MA, United States; 3 MedLink Global, Inc, San Francisco, CA, United States; 4 University of Pennsylvania, Philadelphia, PA, United States; 5 Department of Child and Adolescent Psychiatry, Charité - Universitätsmedizin Berlin, Berlin, Germany; 6 Einstein Center Population Diversity (ECPD), Berlin, Germany; 7 Einstein Center for Youth Mental Health (ECYM), Berlin, Germany; 8 The Zucker Hillside Hospital, Northwell Health, Hempstead, NY, United States; 9 The Feinstein Institute for Medical Research, Center for Psychiatric Neuroscience, Manhasset, NY, United States; 10 German Center for Mental Health (DZPG), Partner Site Berlin, Berlin, Germany; 11 German Center for Child and Adolescent Health (DZKJ), Partner Site Berlin, Berlin, Germany

**Keywords:** comorbidity, general psychopathology factor (p-factor), hierarchical taxonomy of psychopathology (HiTOP), multidimensional psychological assessment, psychopathology, transdiagnostic dimensional models

## Abstract

Traditional diagnostic systems, such as the DSM-5 and ICD-11, suffer from well-documented limitations, including high comorbidity rates, arbitrary diagnostic thresholds, and within-disorder heterogeneity. Consequently, the field is evolving towards hierarchical, transdiagnostic, dimensional models of psychopathology. This study reports on the development and initial validation of the Multidimensional Transdiagnostic Inventory of Psychopathology (MTIP). This new broadband inventory aligns traditional DSM disorders with contemporary dimensional models of psychopathology. We conducted initial development and basic psychometric analyses in a mixed sample of 301 adults. We then validated the MTIP in a second independent sample (N = 826), of which 640 who self-reported no history of past mental health treatment were used as our normative/reference sample. The final MTIP contained 13 clinical scales and seven supplemental scales. All the MTIP scales met core psychometric assumptions at the item, scale, and transdiagnostic levels. A series of exploratory and second-order factor analyses supported the MTIP hierarchical dimensional structure. Concurrent validity correlations with the DSM-5 level 1 cross-cutting symptom measure were strong, ranging from 0.89 (depression) to 0.51 (drug misuse). Regression analyses supported the construct validity of the higher-order transdiagnostic scales. Twelve of the 13 clinical scales differentiated individuals with a history of mental health care from those without past care, with effect sizes ranging from 0.20 (Alcohol Misuse) to 1.06 (Anxiety). Effect sizes for the transdiagnostic scales ranged from 0.33 (Thought Dysregulation) to 1.07 (Emotion Dysregulation). The clinical implications and potential applications of the MTIP are discussed.

## Introduction

Traditional diagnostic systems, such as the Diagnostic and Statistical Manual of Mental Disorders (DSM-5; American Psychiatric Association, 2013) and the ICD-11 (World Health Organization, 2019), define mental disorders as distinct entities or natural kinds (Blashfield, 1984). However, research has revealed considerable non-random comorbidity among clusters of DSM disorders (Krueger and Markon, 2006a; Kessler et al., 2005; Kessler et al., 2011). What began as an exploration of the “problem” of comorbidity evolved into broader research on the underlying structure of psychopathology (Ofrat and Krueger, 2012) and gave rise to contemporary quantitative models.

Contemporary models reorganize traditional DSM disorders and symptoms into a few hierarchically arranged, interrelated transdiagnostic dimensions. Instead of discrete diagnoses, these models conceptualize psychopathology as continuous symptom dimensions that cut across traditional disorders (Krueger and Markon, 2006a). The discovery that major depression and generalized anxiety are components of a higher-order structure explains their frequent comorbidity. These disorders tend to co-occur because they share the same underlying vulnerability (Krueger and Markon, 2006b). Early studies (Krueger, 1999) showed that common psychiatric disorders could be organized into two broad transdiagnostic dimensions: Internalizing (containing unipolar depression, generalized anxiety, social phobia, and panic disorder) and Externalizing (containing alcohol abuse, drug abuse, and adult antisocial behavior).

When additional psychiatric conditions (i.e., psychotic experiences, mania, and paranoia) were included, further dimensions emerged. Wright et al. (2013) found three (3) transdiagnostic dimensions: Internalizing, Externalizing, and Psychosis. Kotov et al. (2011b) explored 25 DSM disorders and identified five transdiagnostic dimensions: Internalizing, Externalizing, Thought Disorder, Somatoform, and Antagonism. Likewise, Markon (2010) modeled a broad range of symptom-level data, including personality disorder symptoms, and identified four transdiagnostic dimensions: Internalizing, Externalizing, Thought Disorder, and Pathological Introversion. Although the number of transdiagnostic dimensions has varied across studies, the Internalizing, Externalizing, and Thought Disorder dimensions have been widely replicated across diverse samples, data, and methods, suggesting that they form core features of psychopathology (Caspi et al., 2014; Forbes et al., 2026; Kim and Eaton, 2015; Kotov et al., 2011a; Krueger and Markon, 2006a; Wright et al., 2013).

Furthermore, the transdiagnostic dimensions are moderately intercorrelated, suggesting the presence of an overarching general factor of psychopathology. This general or *p*-factor (Caspi and Moffitt, 2018; Lahey et al., 2012) reflects the positive correlations among psychiatric disorders and is conceptually similar to Spearman’s general factor of cognitive ability (g-factor, Spearman, 1904). Although its meaning remains debated (see Hyland et al., 2020; Watts et al., 2020), the p-factor appears to summarize an individual’s overall psychiatric severity or burden (Lahey et al., 2021). The *p-factor is statistically robust, replicated across samples and measurement methods, and* stable over time (Lahey et al., 2021; Snyder et al., 2017). High standing on the *p*-factor has been associated with greater functional impairment (Waszczuk et al., 2017), higher comorbidity and risk of relapse (Lahey et al., 2012), and suboptimal treatment response (Caspi and Moffitt, 2018). Genetic research also supports the existence of the *p*-factor, as Grotzinger et al. (2025) identified an overarching genetic liability to a wide variety of psychiatric disorders. These findings have important implications for how psychopathology is conceptualized, assessed, and diagnosed. Smith et al. (2020) argued that clinical assessment would benefit from instruments grounded in contemporary models of psychopathology, especially those that assess the *p*-factor.

By reorganizing DSM disorders into a few interrelated dimensions, transdiagnostic models offer a more parsimonious approach to studying and assessing mental illnesses (Kotov, Ruggero, Krueger, et al., 2011b). Regarding assessment, dimensional measures are more reliable than categorical measures, suggesting greater validity (Price, 2017). A meta-analysis by Markon et al. (2011) found a substantial (37%) increase in validity for dimensional measures of psychopathology over traditional categorical measures. It has also been argued that employing transdiagnostic dimensions in research will enhance our ability to identify etiological factors, such as neurobiological correlates of mental illness (Ofrat and Krueger, 2012), including genetic vulnerabilities (Kendler et al., 2003). The findings from a recent large-scale psychiatric genomics study support this argument. Grotzinger et al. (2025) found that five underlying genomic factors accounted for the majority of the genetic variance in 14 individual psychiatric disorders. Three of the five genomic factors identified in the study – anxiety, depression, and PTSD (internalizing factor); schizophrenia and bipolar disorders (thought disorder factor); and substance use disorders involving a combination of alcohol, cannabis, opioid, and nicotine misuse (externalizing factor) – are similar to the core dimensions of psychopathology described above (internalizing, externalizing, and thought disorder). Grotzinger et al. (2025) also identified compulsive disorders and neurodevelopmental conditions. With dimensional models demonstrating their influence in basic and applied research (Cuthbert, 2005; Hyman, 2010; Hyman, 2011), there is a growing need for psychometrically sound clinical assessment tools that align with these contemporary models.

Current popular psychological inventories, such as the Minnesota Multiphasic Personality Inventory (MMPI-2; Butcher et al., 1989) and the Personality Assessment Inventory (PAI; Morey, 2007), were developed prior to the emergence of transdiagnostic models of psychopathology. Research has shown that these broadband instruments can capture, to varying degrees, the transdiagnostic dimensions reviewed above. For instance, the PAI full-scale factor structure identifies the Internalizing and Externalizing dimensions of psychopathology (Hopwood and Moser, 2011). The most recent version, the MMPI-3, includes Internalizing, Externalizing, and Thought Disorder scales (Ben-Porath and Tellegen, 2020) but lacks a dedicated p-factor scale. However, while similar dimensions can be identified or created from existing inventories, these instruments were not designed to replicate the hierarchical structure of transdiagnostic models, including the overarching *p*-factor.

Based on the extensive and well-replicated findings reviewed above (Caspi et al., 2014; Forbes et al., 2026; Kim and Eaton, 2015; Krueger and Markon, 2006a; Wright et al., 2013) and the emerging genetic evidence (Grotzinger et al., 2025), three core dimensions of psychopathology can be identified: Internalizing, Externalizing, and Thought Disorder. These three dimensions encompass the majority of psychiatric disorders typically treated by mental health professionals (Kotov et al., 2011b; Wright et al., 2013). In addition, the three dimensions are intercorrelated and, when factor analyzed, yield a meaningful overarching *p*-factor. This generic three-dimensional hierarchical model offers a solid conceptual and empirical foundation for developing modern psychological inventories.

One major benefit of hierarchical dimensional assessment is its ability to handle comorbidity. It is well known that psychiatric patients often have multiple disorders and subclinical conditions. By assessing psychopathology at multiple levels, an inventory can capture primary conditions while also identifying additional disorders or subclinical presentations. This approach gives clinicians a complete profile of the individual’s psychiatric status. For example, if a patient’s ADHD caused them anxiety, they would be expected to have some elevation, perhaps subclinical, on the anxiety scale. In addition, at the second level of hierarchical assessment, all clinical and subclinical conditions (measured by the inventory) are grouped into their corresponding dimensions, yielding an overall assessment of within-domain psychopathology (Internalizing, Externalizing, and Thought Disorder). The clinician can now assess the extent of related symptoms (as measured by the test) and overall psychiatric burden through the general factor.

To date, only two assessment inventories, the *SPECTRA*: Indices of Psychopathology (Blais and Sinclair, 2018; Blais and Sinclair, 2019) and the HiTOP-SR (Simms et al., 2022), employ the hierarchical transdiagnostic measurement model. The *SPECTRA* is among the briefest broadband measures (96 items). It uses 12 clinical conditions to generate the three core dimensions of psychopathology (Internalizing, Externalizing, and Thought Disorder) and the overarching *p*-factor. The *SPECTRA* replicates the hierarchical dimensional model but, with only three supplemental scales, offers limited coverage of clinical conditions that do not fit cleanly within it.

The HiTOP-SR (self-report) is the most comprehensive hierarchical inventory available. It employs 405 items (more than either the PAI or MMPI-3) to generate 76 primary construct scales covering six dimensions of psychopathology, integrating personality traits and psychopathology (Simms et al., 2022). The HiTOP-SR presents a comprehensive assessment of psychopathology organized within the hierarchical dimensional model. The HiTOP-SR has only recently become available (on the consortium’s website), leaving its clinical utility unclear. Its length and organizational complexity may limit its clinical application. The HiTOP group has also developed a brief tracker version, the HiTOP-BR. The HiTOP-BR uses 45 items to assess the six HiTOP dimensions and is recommended as a follow-up measure or brief screening tool. Because it assesses only the higher-order dimensions, it lacks the granular information needed to formulate a clinical diagnosis or a tailored treatment plan.

Given the growing acceptance and influence of transdiagnostic dimensional models of psychopathology (Conway et al., 2019), a low-burden psychological inventory is needed that provides a multi-level transdiagnostic assessment of psychopathology based on familiar DSM conditions, along with supplemental scales assessing several clinical syndromes that do not fit neatly within the core dimensions of psychopathology and constructs associated with the broader focus on meaningful mental health outcomes (Lousada, 2015; Zimmerman et al., 2006).

This paper reports on the development and initial validation of the Multidimensional Transdiagnostic Inventory of Psychopathology (MTIP), a proprietary instrument of MedLink Global, Inc. MTIP is the assessment component of MaiNDS, an AI-augmented measurement system that, when integrated with the MTIP, will include an AI-conducted post-testing profile review and exploration. While under development, the goal of the post-testing AI follow-up is to explore how mental health issues affect the individual’s life (to contextualize the findings) and to help establish the degree of distress and functional impairment being experienced. Distress and impairment are important components of an evaluation and often cannot be determined from test findings alone. In fact, research suggests that symptom severity (total symptom count) and functional impairment are only moderately correlated (e.g., r’s between 0.43 and 0.63; Kroenke et al., 2001; Spitzer et al., 2006) and represent semi-independent components of overall mental health (Ro and Clark, 2014). The AI follow-up is intended to improve the MTIP’s assessment of impairment and contextualize the test findings.

The MTIP is intended to be a relatively low-burden inventory that reorganizes 13 familiar DSM syndromes into three core, higher-order dimensions (Internalizing, Externalizing, and Thought Disorder), generates an overarching p-factor, along with a range of treatment-relevant supplemental scales. Thus, it provides an integrated assessment of psychopathology across three levels of specificity: syndrome, transdiagnostic, and global. In addition, it also assesses Body Distress, Eating Issues, Cognitive Complaints, Suicidal Ideation, Personality Dysfunction, Resilience, and Treatment Attitude to enhance clinical formulation, treatment planning, and progress monitoring.

## Study I: development

### Methods

Study I aimed to identify lower-order constructs as markers for the core dimensions, generate an initial item pool, and conduct an initial psychometric evaluation.

### Procedures

These studies did not obtain institutional ethical review. However, the data were all collected anonymously, and all data handling and storage complied with the ethical standards of the American Psychological Association. To develop the MTIP, we identified familiar DSM constructs as markers (syndrome-level scales) for the three core Transdiagnostic Scales (core dimensions of psychopathology). Previous research supports using DSM symptoms and disorders as markers for dimensional models of psychopathology (Forbes et al., 2025; Kotov et al., 2011b). Potential constructs were selected based on clinical utility (e.g., prevalence rates and severity) and their degree of representation in the empirical literature. Candidate markers for the Transdiagnostic Emotion Dysregulation scale (TED, Internalizing) are well established in the literature (see Blais and Sinclair, 2018; Kotov et al., 2011a; Krueger, 1999; Snyder et al., 2023; Wright et al., 2013): Depression (Dep: defined as depressive affect and ideation); Generalized Anxiety (Anx: defined as worry and fear of losing control); Panic (Pan: defined as physiological fear and reactivity); Social Anxiety (SocA: defined as social avoidance and social inadequacy) and Traumatic Stress (Ts: defined as intrusive memories, avoidant behavior, and physical reactivity related to a stressful event). Candidate markers for the Transdiagnostic Behavior Dysregulation scale (TBD, Externalizing) were identified based on prevalence and relevant literature (Blais and Sinclair, 2018; Krueger et al., 2021; Venables and Patrick, 2012; Wright et al., 2013): Alcohol Abuse (Alc: defined as social or personal problems related to alcohol use); Drug Abuse (Drg: defined as a preoccupation with and problems caused by drug use); Antisocial Traits (Ant: defined as disregard for social, ethical or legal convention); Impulsivity (Imp: defined as behavioral dysregulation and lack of planning) and Anger/Aggression (Ang: defined as poorly controlled explosive anger and violence). Markers for the Transdiagnostic Thought Dysregulation scale (TTD, Thought Disorder) were identified based on severity and the relevant research literature by Blais and Sinclair (2018), Conway et al. (2021), Kotov et al. (2024), Kotov et al. (2016), Markon (2010), and Wright et al. (2013). Across these studies, Psychotic Perception (Psy: defined as visual and auditory misperceptions) and Paranoid Ideation/Paranoid Personality (Par: defined as ideas of reference and persecution) were consistently associated with the TTD dimension. While the placement of bipolar illness in dimensional models is debated (Conway et al., 2021), there is strong empirical support for including manic activation/acceleration (Man: defined as mental and physical acceleration) in the TTD dimension (see Blais and Sinclair, 2018; Caspi et al., 2014; Kotov et al., 2011a; Kotov et al., 2016; Kotov et al., 2024; van Os and Reininghaus, 2016). Recent genomic findings (Grotzinger et al., 2025) also support placing mania within the TTD dimension. Candidates for a fourth TTD marker were less clear. Four markers were desired to maintain a balanced representation across the core dimensions. Grandiose Ideation (Grd: defined as an elevated sense of importance, talent, and destiny), while less prominent in the literature, was selected to capture the elevated mood and self-esteem associated with mania and potentially delusions of grandeur. Factor analytic studies of the *SPECTRA* support the placement of manic activation and grandiose ideation on the Thought Disorder dimensions (Blais and Sinclair, 2018; Blais et al., 2021). Lastly, in a large multinational sample, Quattrone et al. (2019) found the manic criteria of “increased self-esteem” and “grandiose delusions” both loaded onto a general factor of psychosis.

This process identified 13 potential markers (disorders or syndromes) covering the three Transdiagnostic Dimensions. To enhance the clinical utility, MTIP includes supplemental scales assessing Body Distress (Bdy: defined as non-specific somatic complaints), Eating Issues (Eat: defined as preoccupation with food or eating), Cognitive Complaints (Cog: defined as subjective cognitive dysfunction), Personality Dysfunction (PD: defined as an underdeveloped sense of self and others), Resilience (Rsl: defined as personal agency and well-being), Treatment Attitude (TxA: defined as optimism and openness to treatment), and Suicidal Ideation (Si: defined as thoughts of death and dying) that were also developed in conjunction with the MTIP transdiagnostic scales. Supplementary Table S1 summarizes the MTIP’s measurement model map (Price, 2017).

Item Pool Development. The initial items were written primarily by the first author (MB), drawing on his background in test development (Baer et al., 2000; Blais et al., 1999; Blais and Sinclair, 2018; Hilsenroth et al., 2005) and item-level research (Blais et al., 2001a; Blais et al., 2001b; Siefert et al., 2009). Broadband proprietary inventories, SPECTRA (Blais and Sinclair, 2018) and the Personality Assessment Inventory (Morey, 2007), were reviewed for content only. Construction of the item pool was guided by the construct definition (see above), the DSM criteria, and a review of common brief inventories. The inventories were compared to each other and the DSM-5 criteria. Areas of overlap among the inventories and the DSM-5 criteria helped identify key features of the target disorder and guided item development. The items were written as brief, focused statements targeting a specific behavior, emotion, or experience. Other project members were consulted as needed regarding item generation. This process generated an initial pool of 150 items covering the clinical and supplemental scales. See Supplemental Table S1 for a list of material consulted during the item pool development.

Three outside experts, all experienced clinicians, and one project member (CC), a recognized content expert, reviewed the item pool. Reviewers assessed each item for clarity, redundancy, and construct relevance. They flagged any item that raised concerns in any of these areas. The first author reviewed the ratings. Items were identified for removal if two or more raters raised concerns about any criterion. Based on this review, 15 items were removed, leaving an initial pool of 135 items. Redundancy and clarity were the main reasons items were flagged. Items were set to a 5-point response scale (not at all true, a little true, moderately true, quite a bit true, and extremely true). Given the expected brevity of the MTIP scales (4 to 6 items each), the five-point response scale seemed the best option to increase potential variance without sacrificing response accuracy or discrimination (Streiner and Norman, 2003).

### Psychometric evaluation and refinement

*Recruitment*: The 135-item MTIP beta version (*MTIP-B)* was evaluated in a sample recruited through the Prolific Academic crowdsourcing platform. Prolific is a website platform that enables researchers to recruit participants for online research. Unlike earlier crowdsourcing platforms, such as Amazon Mechanical Turk (MTurk), Prolific primarily focuses on academic and marketing research. Prolific actively screens and rates participants’ performance to improve and maintain data quality. Studies examining data quality across web platforms have shown that Prolific provides high-quality data (Douglas et al., 2023). Thalmayer et al. (2023) used the prolific platform to recruit the validation sample for the International Mental Health Assessment Inventory (see Study 3). Lipsitz et al. (2026) also used a Prolific-drawn sample to explore the latent structure of psychopathology and neurocognitive functioning. Subjects were required to be 18 years or older, live in the United States, and be fluent English speakers. Subjects read a general description of the study prior to taking the survey. Continuing the survey was taken as informed consent. The platform set the reimbursement rate. The format required subjects to answer each question before moving on. As a result, there were no missing data. The survey included four attention checks placed at roughly equal intervals throughout.

*Participants*: The development sample consisted of 301 adults. The mean age of the development sample was 42.8 (SD = 13.8; range, 19–76), and slightly more than half (56%) were female. Most participants identified as Caucasian (80%) and had at least a high school education (81%). The sample contained 99 subjects (33%) who self-reported no past mental health treatment, 110 (36%) reported past mental health treatment, and 92 (31%) were currently receiving mental health treatment. All study participants completed the *MTIP-B* via the prolific website anonymously after providing informed consent.

*Data analytic approach*: We evaluated the psychometric properties of the MTIP-B using an approach similar to that used to develop other broadband measures of psychological functioning and personality (see Blais and Sinclair, 2018; Morey, 2007). We used Principal Component Analysis (PCA) in the beta phase rather than true factor analysis due to sample size considerations and the goal of identifying linear combinations of items that form scales within each transdiagnostic dimension (Thompson, 2004). First, a series of PCAs was conducted to assess the association between the items and the intended scales. For example, all the Emotion Dysregulation items were analyzed to determine their association with the target scales (e.g., depression, anxiety, social anxiety, and traumatic stress). Items were retained if they had a primary loading of 0.40 or greater on the target scale and no secondary loadings within 0.10 of the primary loading. We set the boundary for a meaningful loading at 0.30. Loadings below 0.30 were not reported (Thompson, 2004). PCAs with Promax rotation were conducted for each transdiagnostic item pool. Items that satisfied these criteria were retained and assigned to their corresponding target scales. Next, we assessed internal consistency reliability (coefficient alpha), adjusted item-to-scale correlations, and mean inter-item correlations for all the MTIP scales. Next, a scale-level PCA was used to provide an initial assessment of the MTIP higher-order structure. All analyses were conducted using SPSS Statistics 24 (IBM Corp, 2016) and FACTOR (Lorenzo-Seva and Ferrando, 2006).

### Results: psychometric evaluation of the MTIP-B

Based on the PCA results, the TED dimension contained four scales and 21 items (Dep k = 6, Anx k = 7, SocA k = 4, and Ts k = 4). The anxiety and panic items failed to separate and were combined to form a single scale. The TBD dimension contained five scales and 23 items (Alc k = 4, Drg k = 4, Ang k = 5, Imp k = 5, and Ant k = 5). The TTD dimension contained four scales and 18 items (Psy k = 5, Par k = 5, Man k = 4, and Grd k = 4). The internal consistency (alpha) for the 13 scales was generally strong, ranging from 0.94 (Anx) to 0.64 (Psy), with a mean of 0.79 and a median of 0.81. Only the Psy scale yielded an alpha below 0.70, the commonly accepted lower boundary for group-level comparisons (Nunnally and Bernstein, 1994). All adjusted item-to-scale correlations exceeded *r* = 0.30, the accepted lower boundary (Nunnally and Bernstein, 1994). Mean item intercorrelations ranged from 0.69 (SocA) to 0.29 (Psy), with a mean of 0.49 and a median of 0.46.

*MTIP-B Scale level structure*: The PCA of the 13 scales revealed three components with Eigenvalues greater than 1.00 (C-1 = 4.91, C-2 = 2.15, and C-3 = 1.09). The three components accounted for 62.66 percent of the total variance. Primary loadings were defined as values of at least 0.40 on the target scale and exceeding the next-highest loading by 0.10 or more. Secondary loadings below 0.30 were not reviewed. Component 1 had loadings for all the Emotion Dysregulation Scales (Anx, SocA, Dep, and Ts), ranging from 0.92 (Anx) to 0.84 (Ts). Component 2 had loadings for all the Behavior Dysregulation scales (Alc, Drg, Ant, Imp, and Ang), with loadings ranging from 0.87 (Alc) to 0.45 (Ang). Component 3 had primary loadings for all the Thought Dysregulation scales (Psy, Par, Man, and Grd), with loadings ranging from 0.95 (Grd) to 0.49 (Man). Only two scales had cross-loadings ≥0.30, the boundary set for meaningful loadings (Thompson, 2004): The Manic Activation scale (Man) loaded onto C-2 (Behavioral Dysregulation) at 0.3, and Grandiose Ideation (Grd) loaded onto C-1 (Emotional Dysregulation) at −0.38. Research supports the cross-loading of Man with Behavioral Dysregulation (see Conway et al., 2021), while the negative cross-loading of Grd onto Emotional Dysregulation is consistent with clinical expectations (e.g., low self-esteem in patients with mood and anxiety disorders). Component intercorrelations ranged from 0.31 (C-1 & C-3) to 0.49 (C-2 & C-3).

*Group differentiation*: of the 301 subjects in the development sample, 99 self-reported having no current or past mental health treatment, and 92 self-reported that they were currently in mental health treatment. Independent *t*-tests showed that the treatment group scored significantly higher (*p* < 0.05) on 12 of the 13 clinical scales. Only the Anger scale showed no significant group difference.

*Supplemental scales*: the internal consistency (alpha) for the seven supplemental scales was generally strong, ranging from 0.85 (PD) to 0.69 (TxA), with a mean of 0.79 and a median of 0.82. All adjusted-item-to-scale correlations exceeded *r* = 0.30. The mean item intercorrelations for the scales ranged from 0.67 (Si) to 0.36 (TxA), with a mean of 0.48 and a median of 0.47.

Based on the analyses reported above, the MTIP item pool was reduced to 92 items covering 13 clinical scales, 7 supplemental scales, and a three-item validity scale.

## Study II: validation

### Methods

*Concurrent validity measure*: DSM-5 Level 1 Cross-Cutting Symptom Measure (DSM-XC) was used to assess the concurrent validity of the MTIP scales. The DSM-XC is a brief, comprehensive screening tool developed to screen for a wide range of common and severe DSM-5 mental disorders (American Psychiatric Association, 2013). Maintaining a connection with familiar DSM disorders was an important goal in developing the MTIP. Although the dimensional model aims to replace the DSM, most research on it has used DSM-defined constructs. The research has consistently shown that DSM symptoms and disorders can be reorganized to reflect the core dimensions of psychopathology (Conway et al., 2019; Forbes et al., 2025; Kim and Eaton, 2015). Based on these considerations, we selected the DSM-XC as a concurrent validity measure to provide initial evidence of construct validity and assess the extent to which the MTIP low-order (syndrome) scales corresponded with related DSM disorders. In addition, the DSM-XC’s brevity was a positive feature, as it did not impose an excessive burden on participants.

The DSM-XC assesses 13 psychiatric syndromes: depression, anger, mania, anxiety, somatic symptoms, suicidal ideation, psychosis, insomnia, memory, repetitive thoughts and behaviors, dissociation, personality functioning, and substance use. The DSM-XC has demonstrated convergent validity with commonly used measures, including the Patient Health Questionnaire for Depression (PHQ-9) and the Penn State Worry Questionnaire, and has shown acceptable to good internal consistency in several studies with adult and college samples (Doss and Lowmaster, 2022; Roche et al., 2019).

The original DSM-XC contains 23 items that ask participants to indicate how much or how often they have been bothered by each problem during the past two weeks. Participants rate the items on a five-point scale: not at all (none), rare, less than a day or two (slight), several days (mild), more than half the days (moderate), and nearly every day (severe). Many of the DSM-XC items contained multiple syndrome indicators (e.g., item 20, “Not feeling close to others or not enjoying your relationships with them?”). For use in this study, these compound items were uncoupled. For example, item 20 was rewritten as two items: “Not feeling close to others” and “Not enjoying my relationships.” This modification increased the DSM-XC to 33 items, which were set on the same 5-point rating scale used by the MTIP (ranging from “Not at all true” to “Extremely true”).^1^ Construct validity of the transdiagnostic scales was explored using a series of stepwise multiple regression analyses (SMRA), calculating multiple R and adjusted R^2^ (adjusting for the number of covariates in the final model).

*Participants*: The revised MTIP and modified DSM-XC produced a combined inventory of 140 items. We fielded the combined MTIP inventory in a second, independent sample (participants from the development sample were not eligible for this study). The validation sample consisted of 852 participants recruited via the prolific platform. Participants were required to reside in the United States and meet standard eligibility criteria for online behavioral research (e.g., a prior task approval rate ≥95%). The platform set the reimbursement rate. The format required subjects to answer each question before moving on. As a result, there were no missing data. The survey included four attention checks at roughly equal intervals throughout. Subjects read a general description of the study prior to taking the survey. Continuing the survey was taken as informed consent. In addition to demographic questions, participants were asked to indicate if they were currently taking psychiatric medication, currently in mental health treatment, or in the past they had taken psychiatric medication or had been in mental health treatment. A review of response times and the attention checks identified 26 subjects with questionable data (3% of the sample). After removing these data, 826 subjects remained for analysis. Of the 826 subjects who satisfied the retention requirements, 640 self-reported no history of mental health treatment, while 186 self-reported a history of past or current treatment or use of psychiatric medication. We used the 640 (no self-reported treatment group) as the non-patient reference sample. The reference sample was 54% female. Race/Ethnicity: 66% identified as Caucasian, 15% as African Americans, 8% as Asian, 6% as Latino, and 5% endorsed Other. The mean age was 40.39 (SD = 12.88), ranging from 18 to 86. Education level: 1% had less than a high school diploma, 16% were high school graduates, 32% had some college, 36% were college graduates, and 15% had postgraduate education. The majority were employed, either full-time (56%) or part-time (18%), 20% were unemployed, and 6% were retired. Approximately half were married or partnered (48%), 41% were single, and 11% were divorced or separated. Income: 23% reported an annual income of 29 k or less, 42% reported 30-74 k, 14% reported 75-99 k, and 20% reported 100 k or greater. The sample’s geographic distribution was 20% Northeast, 19% Midwest, 40% South, and 21% West.

### Results

We then administered the 140-item combined MTIP-DSM-XC on the prolific platform. Using the reference sample (*n* = 640), we conducted a series of Exploratory Principal Component Analyses (PCA) with Promax rotation to assess how the items associated with the intended scales. For example, all the TED items were analyzed to determine their association with the target scales (e.g., depression, anxiety, social anxiety, and traumatic stress). The TED items yielded four components with Eigenvalues greater than 1.00. All the anxiety items loaded on C-1 (Eigenvalue 10.00) (loadings between 0.95 to 0.70), all the depression items loaded on C-2 (Eigenvalue 1.64) (loadings between 0.89 to 0.52), all the social anxiety items loaded on C-3 (Eigenvalue 1.44) (loadings between 0.91 to 0.79), and all the traumatic stress items loaded on C-4 (Eigenvalue 1.18) (loadings between 0.86 to 0.47). Two Ts items had secondary loadings > 0.30 on the Anx scale; the largest was 0.45 (primary loading: 0.58).

The PCA of the TBD items yielded five components with Eigenvalues > 1.00. C-1 (Eigenvalue 7.02) contained all the drug misuse items (0.90 to 0.81), C-2 (Eigenvalue 2.19) contained all the anger/aggression items (0.90 to 0.70), C-3 (Eigenvalue 1.79) contained all the alcohol misuse items (0.90 to 0.72), C-4 (Eigenvalue 1.60) contained all the impulsivity items (0.84 to 0.54), and C-5 (Eigenvalue 1.16) contained all the antisocial items (0.84 to 0.56). Only three TBD items had secondary loadings ≥ 0.30, the largest of which was 0.35. Two Impulsivity items (Imp) loaded onto the Anger scale (Ang) at 0.32 and 0.35, while one Antisocial item (Ant) loaded onto the Drug Misuse scale (Drg) at 0.32. All three items had primary loadings of 0.55 or higher on their target scale.

The TTD items yielded four components with Eigenvalues > 1.00. C-1 (Eigenvalue 5.22) contained all the psychosis items (0.83 to 0.49), C-2 (Eigenvalue 1.92) contained all the paranoia items (0.89 to 0.52), C-3 (Eigenvalue 1.50) contained all the grandiosity items (0.82 to 0.58), and C-4 (Eigenvalue 1.24) contained all the manic activation items (0.77 to 0.51). Three TTD items, one each from manic activation, grandiosity, and paranoia, had secondary loadings ≥ 0.30 on C-1 (psychosis). The manic activation and grandiosity items had clear primary loadings on their target scale. However, the paranoia item, “I believe certain people are planning to harm me,” had essentially equal loadings on its target scale (0.52) and C-1 (psychosis, 0.51). Scale length considerations and a review of the adjusted item-to-scale correlations supported assigning the item to the paranoia scale. Overall, the PCA findings showed that all items had their primary (or highest) loadings on the intended scale. Only eight of the 60 items (13%) had secondary loadings ≥ 0.30, and only one item lacked primary loading.

Tables 1, 2 summarize the internal consistency reliability (coefficient alpha), adjusted item-to-scale correlations, and mean inter-item correlations for all the MTIP scales. For the 13 clinical scales, the internal consistency ranged from 0.68 (Ant) to 0.91 (Anx), with a median of 0.80. All but two scales, antisocial (0.68) and manic activation (0.69), met or exceeded the acceptable lower boundary of 0.70 (Nunnally and Bernstein, 1994). Adjusted item-to-scale correlations were above *r* = 0.30 for all scales (e.g., Nunnally and Bernstein, 1994). Reliability is inversely related to test length; scales with fewer items have artificially lower reliability (Price, 2017). This matters because the MTIP clinical scales contain 4 to 7 items. Using the Spearman-Brown prophecy formula, we re-estimated the reliability of the antisocial and manic activation scales, assuming each had 10 items. The estimated reliabilities for the 10-item antisocial scale and the manic activation scale were 0.79 and 0.81, respectively.

**Table 1:** Psychometric evaluation of the MTIP: validation sample (*N* = 640).

Clinical scale	Alpha^a^	K^b^	Inter r^c^	Adj Item r^d^
Depression	0.89	6	0.59	0.66–0.85
Anxiety	0.91	7	0.61	0.66–0.85
Social anxiety	0.91	4	0.71	0.79–0.84
Traumatic stress	0.74	4	0.42	0.52–0.69
Alcohol	0.84	4	0.60	0.62–0.78
Drug	0.89	4	0.68	0.67–0.86
Anger	0.82	4	0.55	0.39–0.66
Impulsivity	0.75	5	0.38	0.39–0.63
Antisocial actions	0.68*	5	0.33	0.34–0.57
Psychosis	0.73	5	0.39	0.45–0.59
Paranoid ideation	0.80	4	0.53	0.57–0.71
Manic activation	0.69*	4	0.37	0.46–0.53
Grandiose ideation	0.74	4	0.42	0.46–0.63
Transdiagnostic scales				
TED	0.87	21	0.56	0.52–0.79
TBD	0.77	22	0.39	0.46–0.64
TTD	0.79	17	0.25	0.35–0.56

*Internal Consistency below the lower bound of 0.70; TED = Transdiagnostic Emotion Dysregulation; TBD = Transdiagnostic Behavior Dysregulation; TTD = Transdiagnostic Thought Dysregulation.

a Internal-consistency reliability (Cronbach’s alpha coefficient).

b Number of items.

c Mean inter-item correlation.

d Range of adjusted internal-consistency coefficients.

**Table 2:** Psychometric evaluation of the MTIP: validation sample (*N* = 640).

Supplemental scale	Alpha^a^	K^b^	Inter r^c^	Adj Item r^d^
Body distress	0.70	4	0.37	0.40–0.53
Eating issues	0.71	4	0.39	0.21*–0.66
Cognitive complaints	0.74	4	0.42	0.47–0.65
Resilience	0.72	4	0.39	0.45–0.59
Personality dysfunction	0.78	6	0.38	0.44–0.64
Suicidal ideation	0.68^#^	3	0.44	0.36–0.49
Treatment attitude	0.66^#^	4	0.29	0.26*–0.54

Alpha^a^ = Internal-consistency reliability (Cronbach’s coefficient alpha); K^b^ = Number of items; ^c^ = Mean inter-item correlation; ^d^ = range of adjusted internal-consistency coefficients.

^#^Internal consistency below the lower bound of 0.70; *Adj item correlation below the lower bound of 0.30.

*Impact of demographic variables*: to estimate the influence of demographic variables rigorously, we generated separate regression equations using the demographic variables (age, sex, race, and education) as predictors for each MTIP clinical and transdiagnostic scale. The demographic variables were entered simultaneously into each regression, and the squared partial correlations were calculated for each predictor. The squared partial correlation coefficient provides an estimate of the scale variance accounted for by the predictor (demographic variables) independent of the other predictors. Supplementary Table S2 presents the estimates of variance in MTIP clinical and transdiagnostic scale scores attributable to demographic variables. As shown in the Supplemental Table, the MTIP scales are, for the most part, not meaningfully associated with demographic variables. Age had the greatest impact on MTIP scales, accounting for 2% or more of the variance in Dep (4.2%), Anx (5.9%), SocA (4.2%), Ts (2.1%), Ant (2.9%), and Man (4.5%). Age is known to have an inverse effect on psychopathology (Morey, 2007). Sex affected MTIP scores as expected: Anx (3.2%), with females scoring higher, and Imp (3.2%) and Ant (9.4%), with males scoring higher. Education affected Dep (2.0%), with higher education associated with lower Dep. Race was not meaningfully associated with any MTIP clinical scale (accounting for less than 2 percent of the variance). On average (mean), the demographic variables combined accounted for 5.57% of the variance in the 13 clinical scales (range 1.90 [Alc] to 13.20 [Ant]) and 8.85% of the variance in the transdiagnostic scales (range 5.80 [TBD] to 10.60 [TED]).

Although our findings on the impact of demographic variables align with other broadband assessment inventories, such as the PAI (Morey, 2007) and the SPECTRA (Blais and Sinclair, 2018), the sex difference on the Behavioral Dysregulation scales was somewhat larger than expected. Supplemental Table S3 presents the raw score differences for males and females in the reference sample. As Supplemental Table S3 shows, there were no meaningful differences (effect size, *d* < 0.20) for seven MTIP clinical scales (Dep, SocA, Ts, Ang, Par, Man & Grd). Small effect size differences (*d* > 0.19 and < 0.49) were noted on five clinical scales (Anx, Alc, Drg, Imp, and Psy), and the Ant scale showed a medium difference (d > 0.49 and < 0.79). Effect size differences were most prominent on the Behavioral Dysregulation scales (0.25 to 0.65). The effect size differences for Imp and Ant (0.42 & 0.65) suggest that applying sex-specific norms to these scales, or to all Behavioral Dysregulation scales, may be appropriate.

### MTIP scale level factor structure

Having confirmed the item-level psychometric functioning of the MTIP scales in an independent sample, we next conducted an exploratory principal axis factor analysis (PAF; Thompson, 2004) to evaluate the underlying factor structure of the 13 clinical scales. Our choice of an exploratory rather than a confirmatory factor analysis (CFA) follows the rationale put forth by Hopwood and Donnellan (2010). Hopwood and Donnellan (2010) showed that many commonly used broadband psychological inventories failed to meet standard CFA fit statistics while “performing reasonably well by the more relaxed criteria associated with EFA techniques” (p.340).

The Kaiser–Meyer–Olkin (KMO) (Kaiser, 1974) measure of sampling adequacy was 0.869, and Bartlett’s test of sphericity (*χ*^2^ = 2615.0, *p* < 0.0001, *df* = 78) showed the sample was suitable for factor analysis (Bartlett, 1951). The PAF with an oblique (promax) rotation produced three factors with eigenvalues ≥ 1.00 (Eigenvalues: F-1 = 4.48, F-2 = 1.90, F-3 = 1.19, F-4 = 0.81). The extracted three-factor solution was clear and interpretable, accounting for 49.98% of the total variance. Table 3 presents the Pattern Matrix for the three-factor solution. As shown, the 13 MTIP clinical scales demonstrated a factor structure consistent with the three core transdiagnostic dimensions. All the Emotion Dysregulation scales loaded strongly on F-1 (ranging from 0.95 for Anx to 0.52 for Ts). The five Behavior Dysregulation scales loaded on F-2 from 0.79 (Ant) to 0.38 (Ang), and the four Thought Dysregulation scales loaded on F-3 from 0.74 (Grd) to 0.38 (Man). The MTIP scale loadings were strong (>0.40) and specific for 11 of the 13 scales. Only one scale had a secondary loading > 0.29; Grandiose Ideation loaded at −0.37 on the Emotion Dysregulation factor. The factors showed acceptable internal consistency (Omega; McDonald, 1999): F-1 (TED) = 0.90, F-2 (TBD) = 0.77, and F-3 (TTD) = 0.72. Consistent with the hierarchical dimensional model, the three factors were moderately intercorrelated (factors’ intercorrelations ranged from 0.37 (F-1 & F-3) to 0.61 (F-2 & F-3). The factor scores were saved for use in later analyses.

**Table 3:** EFA principal axis factoring with promax rotation for the MTIP clinical scales.

MTIP clinical scales	Factor 1	Factor 2	Factor 3
Anxiety	**0.95**		
Social anxiety	**0.82**		
Depression	**0.78**		
Traumatic stress	**0.52**		
Antisocial actions		**0.79**	
Impulsivity		**0.76**	
Drug misuse		**0.60**	
Alcohol misuse		**0.48**	
Anger		**0.38**	
Grandiose ideation	−0.37		**0.74**
Psychosis			**0.52**
Paranoid ideation			**0.50**
Manic activation			**0.38**
Factor correlations			
Factor 1	1.00		
Factor 2	0.56	1.00	
Factor 3	0.37	0.61	1.00

*N* = 640. Only loadings ≥ 0.30 are shown. Bold values indicate primary loadings.

Next, the three transdiagnostic scales were constructed by summing the raw scores of the scales that loaded on each factor. For example, the TED scale was created by summing the raw scores for Dep, Anx, SocA, and Ts. We then correlated the manifest transdiagnostic scales with their corresponding factor score. The scale-to-factor correlations were strong (TED: 0.98; TBD: 0.95; TTD: 0.95), indicating that the manifest scales replicate the underlying factors. The Global Psychopathology Severity scale (GPS, p-factor) was created by summing the raw scores for TED, TBD, and TTD. Although some argue that the GPS should be derived from a weighted transformation of the scales, Fried et al. (2021) found that raw scores work equally well.

*Concurrent validity and group differentiation*: Subjects in the validation sample completed the modified DSM-XC measure, from Section III: Emerging Measures and Models of DSM-5 (APA, 2013). As described above, we modified the original 23-item DSM-XC by separating compound items (items with two or more construct indicators), expanding the DSM-XC to 33 items, and using the same 5-point rating scale as the MTIP. The revised DSM-XC generates 11 short scales: depression (4 items [alpha = 0.91]), anxiety (5 items [alpha = 0.87]), mania (3 items [alpha = 0.61]), substance use (3 items [alpha = 0.47]), level of personality (4 items [alpha = 0.82]), psychosis (4 items, including the dissociation item [alpha = 0.81]), anger (2 items [alpha = 0.77]), somatic symptoms (3 items, including the insomnia item [alpha = 0.67]), cognitive problems (2 items [alpha = 0.60]), OCD (2 items [alpha = 0.69]), and suicidal ideation (1 item). Despite their brevity, nine of the 10 DSM-XC scales had alphas > 0.60 and all adjusted item-to-scale correlations > 0.30. The XC-SUD scale, which combines tobacco, alcohol, and drug use, had poor internal consistency and failed to meet the item-to-scale correlation boundary of ≥ 0.30. Despite its weak psychometrics, the XC-SUD scale was included in the construct validity analysis.

Concurrent validity correlations are presented in Table 4. As shown in Table 4, 11 MTIP scales were significantly correlated with, and had their highest correlation with, their corresponding DSM-XC scale. The MTIP Dep, Anx, Psy, and Man scales correlated > 0.70 with their corresponding DSM-XC scales. Three MTIP scales (Alc, Drg, and Ang) correlated > 0.50 with their corresponding DSM-XC scales. The SocA and Ts scales had their highest correlations with the DSM-XC anxiety scale (0.69 and 0.54, respectively). The MTIP Imp scale correlated at 0.46 with the DSM-XC cognitive problems scale, and the MTIP Ant scale had its highest correlation observed here (0.31) with XC-SUD. Only the MTIP Grd scale failed to show a meaningful correlation with the DSM-XC scales. Turning to the supplemental scales, the matched suicidal ideation scales correlated at 0.57, the cognitive complaint scales at 0.64, the somatic (Bdy) scales at 0.72, and the personality functioning scales at 0.82.

**Table 4:** Concurrent validity correlations among the MTIP scales and DSM-5 cross-cutting measure scales.

MTIP	XC-DEP	XC-ANX	XC-SOM
Emotion dysregulation scales			
Dep	**0.89**	0.69	0.56
Anx	0.68	**0.85**	0.53
SocA	0.58	**0.69**	0.41
Ts	0.45	**0.54**	0.40

MTIP	XC-SUD	XC-ANG	XC-COG
Behavior dysregulation scales			
Alc	**0.57**	0.30	0.23
Drg	**0.51**	0.26	0.34
Ang	0.20	**0.58**	0.40
Imp	0.28	0.39	**0.46**
Ant	0.31	0.25	0.23

MTIP	XC-PSY	XC-MAN	XC-COG
Thought dysregulation scales			
Psy	**0.84**	0.34	0.41
Par	0.**43**	0.30	0.34
Man	0.45	**0.71**	0.44
Grd	0.25	0.21	−0.02

N = 640. DSM-XC = DSM-5 Cross-Cutting Measure; DSM-XC scale abbreviations are prefixed with XC-: XC-DEP = Depression; XC-ANX = Anxiety; XC-SOM = Somatization; XC-SUD = Substance Use Disorder; XC-ANG = Anger; XC-COG = Cognitive Concerns; XC-PSY = Psychosis; XC-MAN = Manic Activation. MTIP: Dep = Depression; Anx = Anxiety; Ts = Traumatic Stress; SocA = Social Anxiety; Psy = Psychosis; Par = Paranoid Ideation; Man = Manic Activation; Grd = Grandiose Ideation; Alc = Alcohol Misuse; Drg = Drug Misuse; Ang = Anger/Aggression; Imp = Impulsivity; Ant = Antisocial Actions. Bold = Target scale correlation.

In SMRAs exploring the construct validity of the transdiagnostic scales, the 11 DSM-XC scales were used as predictors, and the MTIP GPS, TED, TBD, and TTD as the dependent variables.

Table 5 presents the final SMRA model for the MTIP GPS scale. Nine of the DSM-XC scales, XC-ANX, XC-PSY, XC-DEP, XC-MAN, XC-ANG, XC-COG, XC-SUD, XC-PF, and XC-OCD, were independent predictors of the MTIP GPS (F [9, 630] = 248.488, *p* < 0.001). The SMRA equation for predicting the GPS scale had a final multiple R of 0.883 and an adjusted *R*^2^ of 0.777.

**Table 5:** Regression coefficients for the final stepwise model for predicting the MTIP global psychopathology severity scale (GPS).

Dependent MTIP GPS	Predictor DSM-XC scales	Unstandardized beta	SE *β*	Standardized *β*	*t*	*p*
GPS	Constant	27.225	1.826		14.906	<0.001
	XC-ANX	1.933	0.180	0.306	10.752	<0.001
	XC-PSY	3.218	0.372	0.190	8.642	<0.001
	XC-DEP	1.094	0.256	0.160	4.276	<0.001
	XC-MAN	1.555	0.263	0.134	5.915	<0.001
	XC-ANG	1.504	0.354	0.105	4.254	<0.001
	XC-COG	1.599	0.469	0.088	3.407	0.001
	XC-SUD	1.133	0.270	0.084	4.200	<0.001
	XC-PF	0.745	0.270	0.097	2.752	0.006
	XC-OCD	1.006	0.487	0.058	2.062	0.039

*N* = 640. GPS = MTIP Global Psychopathology Severity Scale; DSM-XC = DSM-5 Cross-Cutting Measure; DSM-XC scale abbreviations are prefixed XC-: XC-DEP = Depression; XC-ANX = Anxiety; XC-SOM = Somatization; XC-SUD = Substance Use Disorder; XC-ANG = Anger; XC-COG = Cognitive Concerns; XC-PSY = Psychosis; XC-MAN = Manic Activation; XC-PF = Personality Functioning; XC-OCD = Obsessive-Compulsive Disorder; XC-SUI = Suicidal Ideation.

Table 6 presents the final SMRA model for the MTIP TED, TBD, and TTD scales. Six DSM-XC scales, XC-ANX, XC-DEP, XC-COG, XC-SOM, XC-PF, and XC-MAN (negative) were independent predictors of the TED scale (F [6, 633] = 522.865, *p* < 0.001), with a final multiple *R* of 0.912 and an adjusted *R*^2^ of 0.831. Nine DSM-XC scales, XC-OCD, XC-SUD, XC-ANG, XC-MAN, XC-PSY, XC-COG, XC-SUI, XC-SOM (negative), and XC-ANX (negative), were independent predictors of TBD (F [9, 630] = 85.014, *p* < 0.001), with a final multiple R of 0.741 and an adjusted *R*^2^ of 0.542. Two DSM-XC scales, XC-PSY and XC-MAN, were independent predictors of TTD (F [2, 637] = 321.170, *p* < 0.001), with a final multiple *R* of 0.709 and an adjusted *R*^2^ of 0.501. Supplementary Tables S2, S3 present the stepwise regression model summaries for the DSM-XC prediction of the MTIP Transdiagnostic and Global Psychopathology Severity Scales.

**Table 6:** Regression coefficients for the final stepwise model for the prediction of the three MTIP transdiagnostic scales by the DSM-XC scales.

Dependent MTIP	Predictor DSM-XC	Unstandardized *β*	SE *β*	Standardized *β*	*t*	*p*
TED						
	Constant	7.343	0.818		8.975	<0.001
	XC-ANX	2.114	0.096	0.531	21.951	<0.001
	XC-DEP	1.276	0.138	0.296	4.214	<0.001
	XC-COG	0.889	0.256	0.077	3.520	<0.001
	XC-SOM	0.448	0.136	0.070	3.299	0.001
	XC-PF	0.447	0.148	0.092	3.025	0.003
	XC-MAN	−0.404	0.138	−0.055	−2.928	0.004
TBD						
	Constant	10.268	0.882		11.646	<0.001
	XC-OCD	0.989	0.236	0.172	4.198	<0.001
	XC-SUD	1.367	0.129	0.302	10.606	<0.001
	XC-ANG	1.262	0.162	0.265	7.775	<0.001
	XC-MAN	0.756	0.125	0.195	6.036	<0.001
	XC-PSY	0.878	0.178	0.156	4.941	<0.001
	XC-SOM	−0.323	0.116	−0.096	−2.795	0.005
	XC-COG	0.597	0.225	0.098	2.653	0.008
	XC-ANX	−0.208	0.080	−0.099	−2.600	0.010
	XC-SUI	0.824	0.409	0.066	2.014	0.044
TTD						
	Constant	8.411	0.726		11.592	<0.001
	XC-PSY	2.560	0.151	0.510	16.960	<0.001
	XC-MAN	1.167	0.104	0.333	11.27	<0.001

*N* = 640. MTIP TED = Transdiagnostic Emotion Dysregulation; TBD = Transdiagnostic Behavior Dysregulation; TTD = Transdiagnostic Thought Dysregulation; DSM-XC = DSM-5 Cross-Cutting Measure; DSM-XC scale abbreviations are prefixed XC-: XC-DEP = Depression; XC-ANX = Anxiety; XC-SOM = Somatization; XC-SUD = substance use disorder; XC-ANG = Anger; XC-COG = Cognitive Concerns; XC-PSY = Psychosis; XC-MAN = Manic Activation; XC-PF = Personality Functioning; XC-OCD = Obsessive Compulsive Disorder; XC-SUI = Suicidal Ideation.

### Group differentiation

To explore the MTIP’s ability to differentiate self-reported past-treatment from no-treatment subjects, we conducted independent t-tests comparing subjects with a history of mental health treatment (N = 186) and those without (N = 640). Table 7 presents the raw mean scores, *p*-values, and corresponding effect sizes (Cohen’s *d*; Cohen, 1988), along with 95% confidence intervals (CIs). As shown in Table 7, the self-report past-treatment group scored significantly higher on all but the Grd MTIP scale, with effect sizes ranging from 1.06 to 0.20. Three MTIP scales, Dep, Anx, and Ts, had effect sizes that fell within the large range (≥0.80), and three scales (SocA, Imp, and Man) fell within the moderate range (≥0.50–0.79). Six scales (Ang, Psy, Par, Ant, Alc, and Drg) had effect sizes within the small range (≥0.20–0.39). Lastly, the past-treatment group scored significantly higher on all four transdiagnostic scales. The MTIP TED and GPS scales had large effect sizes, while the TBD had a moderate effect size, and the TTD had a small effect size.

**Table 7:** Group differentiation.

MTIP scales	No Tx mean	Past Tx mean	*p*-value	Effect size*	ES 95% CI
Dep	11.79	17.17	<0.001	0.96	0.79–1.13
Anx	12.81	19.63	<0.001	1.06	0.89–1.23
SocA	8.90	11.82	<0.001	0.65	0.48–0.81
Ts	8.46	11.24	<0.001	0.80	0.63–0.97
Alc	4.74	5.17	0.034	0.20	0.04–0.37
Drg	4.45	4.87	0.021	0.23	0.07–0.39
Ang	5.46	6.70	<0.001	0.49	0.32–0.65
Imp	7.38	9.22	<0.001	0.58	0.42–0.75
Ant	8.16	8.99	<0.001	0.29	0.12–0.45
Psy	5.99	6.69	0.003	0.31	0.14–0.47
Par	5.83	6.66	0.003	0.29	0.13–0.46
Man	5.94	7.64	<0.001	0.61	0.44–0.78
Grd	8.21	7.67	0.052	NS	
Transdiagnostic					
TED	41.96	59.39	<0.001	1.07	0.90–1.24
TBD	30.17	34.53	<0.001	0.53	0.37–0.70
TTD	25.98	28.57	0.001	0.33	0.16–0.49
GPS	98.12	122.45	<0.001	0.93	0.76–1.10

Raw score means and effect size differences for the no treatment (*N* = 640) and past treatment (*N* = 186) groups.

*Effect Size (ES) = Cohen’s d. Tx = treatment. Dep = Depression; Anx = Anxiety; Ts = Traumatic Stress; SocA = Social Anxiety; Alc = Alcohol Abuse; Drg = Drug Abuse; Ang = Anger/Aggression; Imp = Impulsivity; Ant = Antisocial Behavior; Psy = Psychosis; Par = Paranoid Ideation: Man = Manic Activation; & Grd = Grandiose Ideation. TED = Transdiagnostic Emotion Dysregulation; TBD = Transdiagnostic Behavior Dysregulation; TTD = Transdiagnostic Thought Dysregulation; and GPS = Global Psychopathology Severity.

#### Hierarchical structure of the MTIP

The MTIP was developed to align with contemporary multi-level hierarchical models of psychopathology. To assess the MTIP’s hierarchical structure, we conducted a second-order Exploratory Maximum Likelihood (ML) factor analysis using FACTOR 9 (Lorenzo-Seva and Ferrando, 2006). The second-order factor analyses employed a promax rotation and the Schmid-Leiman procedure, as recommended by Thompson (2004). Researchers have widely used second-order factor models in personality and psychopathology research (Reise et al., 2010). In second-order factor analysis, researchers first identify the specific factors (dimensions) and then extract a general factor from the intercorrelated specific dimensions. Second-order models are truly hierarchical, and applying the Schmid-Leiman procedure (Schmid and Leiman, 1957) preserves the oblique solution while revealing the data’s hierarchical structure (Thompson, 2004, p. 74). The KMO (Kaiser, 1974) was 0.869. Fit statistics for the ML EFA three-factor model were: RMSEA = 0.049 (90% CI 0.032–0.055), Tucker-Lewis Index (TLI) = 0.96, Comparative Fit Index (CFI) = 0.98, Goodness of fit index (GFI) = 0.99, and Adjusted Goodness of fit index (AGFI) = 0.99. The fit statistics fall within the good-to-excellent range (Hu and Bentler, 1999).

Table 8 shows that the MTIP’s three transdiagnostic dimensions (scales) combine to yield a second-order general component. All 13 clinical scales loaded onto the second-order (general) factor, with a mean loading of 0.45 (range: 0.22–0.68). In addition, all clinical scales remained primarily associated with their target dimension, although the magnitude of the loadings was reduced. The three transdiagnostic dimensions (Factors) loaded on the general factor 0.57 (TED), 0.88 (TBD), and 0.62 (TTD). The overarching general factor showed good internal consistency (Omega = 0.83; Standardized Alpha = 0.83). The Root Mean Square of Residuals (RMSR) = 0.0267 was lower than the expected mean value of RMSR (0.0403), indicating an acceptable model fit (Kelley, 1935).

**Table 8:** Second-order maximum likelihood factor analysis with promax rotation.

Clinical scales	S1 (TTD)	S2 (TBD)	S3 (TED)	G-factor
Anxiety			**0.77**	0.45
Social anxiety			**0.67**	0.34
Depression			**0.64**	0.49
Traumatic stress	0.23		**0.41**	0.43
Antisocial actions		**0.38**		0.57
Impulsivity		**0.34**		0.68
Drug misuse		**0.20**		0.42
Anger		**0.16**		0.53
Alcohol misuse		**0.15**		0.34
Grandiose ideation	**0.57**		−0.31	0.22
Psychosis	**0.45**			0.50
Paranoid ideation	**0.36**			0.44
Manic activation	**0.30**			0.50
			Factor G loadings	
			Factor 1	0.62
			Factor 2	0.88
			Factor 3	0.57

*N* = 640. Only primary loadings and secondary loadings ≥ 0.20 are shown. Bold = Primary Loading; S = Specific Factor; G = General Factor.

## Discussion

The goal of this study was to develop a hierarchical transdiagnostic inventory of psychopathology based on familiar DSM constructs and consistent with contemporary quantitative models. These initial results suggest that the MTIP is a reliable and valid measure for assessing both traditional DSM-based clinical syndromes and the hierarchical transdiagnostic dimensions of psychopathology. In this way, the MTIP provides a parsimonious and comprehensive assessment of psychopathology spanning multiple levels of specificity.

Item-level analyses across the development and validation samples showed that the MTIP items were primarily and (almost) exclusively associated with their target construct (scale). Furthermore, despite their brevity (4 to 7 items), 11 of the 13 MTIP clinical scales showed acceptable internal consistency (above 0.70), while two scales fell just below this threshold (Ant 0.68 and Man 0.69). For all 13 scales, the adjusted item-to-scale correlations exceeded the 0.30 lower boundary. Demographic variables, except age and sex, had minimal impact on the MTIP scales. These findings are similar to those reported for other psychological inventories (see the PAI [Morey, 2007, Table 4.20, p. 97] and the *SPECTRA* [Blais and Sinclair, 2018, Table 4.17, p. 38]). In addition, longitudinal research shows that neuroticism, which is highly correlated with internalizing psychopathology, declines over time (Graham et al., 2020), and a large body of research has documented the role of sex in externalizing conditions (Li et al., 2023). However, the impact of sex on two MTIP Behavior Dysregulations scales, Ant and Imp, was greater than expect and might support using sex specific norms for these two scales. The impact of education was likely underestimated as it was recorded as a categorical variable rather than a continuous one.

The scale-level exploratory factor analyses yielded the expected transdiagnostic structure of Emotion Dysregulation, Behavior Dysregulation, and Thought Dysregulation. A second-order EFA reproduced the three-factor solution and the overarching general factor, with fit statistics falling within the good-to-excellent range. The second-order factor analysis supported the MTIP hierarchical dimensional structure with an overarching *p*-factor at the apex. All 13 MTIP clinical scales loaded onto the general factor and the expected specific factors. The three transdiagnostic factors also had strong loadings on the general factor. Together, the item- and scale-level analyses from two independent samples demonstrate that the MTIP possesses acceptable psychometric properties and accurately replicates the intended hierarchical dimensional measurement model. The manifest transdiagnostic scales (TED, TBD, and TTD) were highly correlated (≥0.95) with their corresponding factor scores. The fit statistics for the second-order factor analyses indicated that the three-factor structure provides an adequate to strong fit to the data (Hu and Bentler, 1999).

Concurrent validity was generally strong (*r* > 0.50, large effect size, Cohen, 1988), with the MTIP scales showing good agreement with their DSM-XC counterparts. The MTIP depression, anxiety, psychosis, and manic activation scales had correlations with their corresponding DSM-XC scale, ranging from 0.71 (Man) to 0.89 (Dep). The MTIP social anxiety, traumatic stress, alcohol, drug, and anger scales correlated with their corresponding DSM-XC scale between 0.51 (Drg) and 0.69 (SocA). Although not directly corresponding, the MTIP impulsivity scale correlated with the DSM-XC cognitive scale (*r* = 0.46), and the MTIP Paranoid ideation scale correlated with the DSM-XC psychosis scale (*r* = 0.43). These concurrent validity correlations support the position that the MTIP scales adequately capture a wide range of traditional DSM constructs (disorders).

The SMRA provides evidence of the construct validity for the higher-order scale. Nine of the eleven DSM-XC scales independently predicted the MTIP GPS, supporting its use as a measure of global psychopathology. In addition, two internalizing scales (XC-DEP and XC-ANX), two externalizing scales (XC-ANG and XC-SUD), and two thought disorder scales (XC-PSY and XC-MAN) predicted the GPS, supporting its breadth of measurement. Internalizing conditions (XC-DEP, XC-ANX, and XC-SOM), cognitive complaints (XC-COG), which commonly co-occur with internalizing disorders (Aschwanden et al., 2020), and personality functioning (XC-PF), which is strongly correlated with negative affect (Krueger and Markon, 2014), predicted the TED scale. TED’s negative association with the XC-MAN scale is clinically and theoretically consistent (Kotov et al., 2011b).

The SMRA findings for the TBD scale were more complex, perhaps because the DSM-XC lacks many externalizing conditions. DSM-XC predictors included the two DSM-XC externalizing disorders (XC-SUD and XC-ANG), two thought disorder scales (XC-MAN and XC-PSY), two internalizing scales (XC-SOM and XC-ANX [negative associations]), and XC-COG and XC-SUI. The thought disorder scales likely reflect the impulsivity (XC-MAN) and eccentricity (XC-PSY) associated with externalizing conditions, while the negative association with the internalizing scales is consistent with the noted lack of anxiety associated with externalizing disorders. A recent study by Faulkenberry et al. (2026) exploring the higher-order structure of the HiTOP-SR (Simms et al., 2022) and MMPI-3 reported similar findings. They found that manic activation (RC9 scale), Cognitive Complaints (COG), and Suicidal Ideation (SUI) were positively associated with the HiTOP-SR’s Externalizing Disinhibition dimension, while the HiTOP-SR’s Externalizing Antagonistic dimension was positively associated with the Aberrant Experiences scale (RC8), and negatively associated with both the somatic complaints (RC1) and the anxiety-related disorders scales (ARX). The association of the XC-OCD scale with the TBD dimension was unexpected. This finding may reflect a limitation of the two-item XC-OCD measure. However, the placement of OCD within the empirical dimensional model has been inconsistent and remains unsettled (Faure and Forbes, 2021). The MTIP TTD scale was exclusively predicted by the two DSM-XC thought disorder scales (XC-PSY and XC-MAN). Taken together, the SMRA findings support the construct validity of the MTIP higher-order scales.

As evidence of the MTIP’s clinical utility, twelve of the 13 MTIP clinical scales and all four transdiagnostic scales significantly differentiated subjects with a self-reported history of mental health treatment from those without such a history. Six MTIP scales had moderate-to-large effect sizes. The one exception was Grandiose Ideation, which did not differentiate the two groups (*p* = 0.052); we discuss possible reasons in the Limitations.

Several limitations need to be acknowledged. Although MTIP’s development process was fairly extensive, these findings require replication in diverse samples with a broader range of concurrent measures (e.g., the HiTOP-BR). In addition, conducting a confirmatory factor analysis in a separate sample would help establish the MTIP factor structure. Moreover, we currently have no test–retest (stability) data. The development sample (*N* = 301) was too small for a simultaneous item-level analysis of the full item pool, so we conducted item-level analyses within each transdiagnostic dimension. The brevity of the MTIP scales (ranging from 4 to 7 items per scale) also raises concerns about the range of construct coverage (e.g., the MTIP depression scale has few items related to physiological features of depression). Future research is needed to more fully explore the sex differences noted on the Behavioral Dysregulation scales. Additional research is needed to better understand the specific features captured by the MTIP scales. The clinical utility analyses showed that the Grd scale failed to differentiate between the no-treatment (standardization sample) and past-treatment groups, with the no-treatment group scoring slightly higher (non-significantly). This finding is not entirely unexpected. Blais and Sinclair (2018) reported that the *SPECTRA* standardization sample scored significantly, although modestly, higher than the clinical sample on the grandiosity scale (see Table 6.16, p. 61). Because the past-treatment group was defined by self-report rather than by verified clinical care, it is not a validated clinical sample, and the present findings likely underestimate the MTIP’s clinical utility. The MTIP is also a proprietary instrument of MedLink Global, Inc.; its items and scoring algorithms are not freely available and require licensing, which constrains independent replication and external validation. Lastly, although the higher-order structure reported here is consistent with contemporary dimensional models of psychopathology, additional research is needed to determine whether the MTIP multidimensional factor structure is invariant across samples.

Despite these limitations, the findings argue that the MTIP provides a sound assessment of 13 important traditional DSM constructs (scales) while capturing the three core transdiagnostic dimensions of psychopathology and the overarching *p*-factor. The findings also provide preliminary support for the MTIP supplemental scales. Given that the DSM-5 (APA, 2013) continues to hold clinical prominence, the correspondence of the MTIP lower-order scales with the DSM is important for clinical continuity. However, the MTIP’s structure aligns with contemporary models of psychopathology, facilitating the translation of new insights and advances, such as the *p*-factor, into clinical practice. Taken together, the MTIP offers a low-burden, psychometrically sound assessment tool that maintains continuity with traditional DSM-5 conditions while capturing the emerging empirical structure of psychopathology. Its brevity and sound psychometrics should make the MTIP a valuable addition to clinical self-assessment tools.

## Data Availability

The original contributions presented in the study are included in the article and supplementary material. The Multidimensional Transdiagnostic Inventory of Psychopathology (MTIP) questionnaire items, scoring algorithms, and the reference datasets analyzed in this study are proprietary to MedLink Global, Inc., and are protected under US Copyright and Patent Pending regulations (Application No. 19/445,513). The datasets, baseline scoring parameters, and standard deviations are not publicly available and are subject to strict licensing restrictions. Inquiries regarding research collaboration or commercial licensing should be directed to MedLink Global, Inc. (mblais@medlink.global).

## Footnotes

1 A copy of the modified DSM-XC items is available from the first author.
