# Supplementary material for: Multidimensional transdiagnostic inventory of psychopathology: development and initial validation
Source: Front Psychol. 2026 Oct 28;17:1917064. doi: 10.3389/fpsyg.2026.1917064 (PMC13751960; doi:10.3389/fpsyg.2026.1917064)
Supplement: Supplementary file 1 [file Supplementary_file_1.DOCX]

**Supplementary Material**

**Multidimensional Transdiagnostic Inventory of Psychopathology: Development and Initial Validation**

**Table of Contents**

|  | **Page** |
| --- | --- |
| **Supplementary Figure 1.** Theoretical foundation and conceptual map for the MedLink Multi-Level Transdiagnostic Inventory of Psychopathology (MTIP) | 3 |
| **Supplementary Table S1.** Item development process: MTIP scales, construct definitions, and materials consulted. | 6 |
| **Supplementary Table S2**. Estimates of Variance in MTIP Clinical and Transdiagnostic Scale Scores Attributable to Demographic Variables | 9 |
| **Supplementary Table S3**. Raw score means and effect size differences by sex (Males, n = 295, and Females, n = 342). | 10 |
| **Supplementary Table S4**. Final Regression Model for the DSM-XC prediction of the MTIP Global Psychopathology Severity Scale (GPS). | 11 |
| **Supplementary Table S5**. Final Regression Models for the DSM-XC prediction of the MTIP Transdiagnostic Scales: TED, TBD, and TTD. | 12 |

**Supplementary Figure 1.** Theoretical foundation and conceptual map for the MedLink Multi-Level Transdiagnostic Inventory of Psychopathology (MTIP)

The MTIP is grounded in the empirical hierarchical dimensional models of psychopathology. These contemporary models organize psychopathology into several distinct, interrelated dimensions that are continuous with normal states and hierarchically arranged. The basic structure of dimensional models has been widely replicated across diverse samples, measurement types (disorders, symptoms, test scales, and items), research methods (longitudinal and cross-sectional), and domains (genetics, neuroscience, epidemiology, and developmental psychology). Furthermore, dimensional models are transdiagnostic in that they focus on the overarching integrated structure of psychopathology rather than individual disorders or syndromes. Hence, the transdiagnostic dimensional approach allows for a more coherent, parsimonious, and multi-level analysis of clinical syndromes and personality traits.

Proposed Structure and Composition.

**Hierarchical Structure and Composition of the MTIP**

The MTIP will assess psychopathology at three levels of specificity, or bandwidth. At the apex will be a global general factor (p-factor)

The three core dimensions of psychopathology will make up the broad mid-level of the hierarchy: Internalizing, Externalizing, & Thought Impairing. A balanced number of lower-order markers will represent the three core dimensions. These markers will comprise more recognizable clinical disorders, maladaptive traits, and symptom clusters.

**Clinical Scales**

**Internalizing Markers**:

**Depression (Dep):** The affective and cognitive features of depression.

**Anxiety (Anx):** Worry and apprehension.

**Social Anxiety (Soc):** Fear of embarrassment in public situations

**Panic (Pan):** Sudden, intense, and unexplained anxiety

**Traumatic Stress (Ts):** Physiological activation and avoidance (cognitive & behavioral) associated with a past trauma/stressful event.

**Externalizing Markers:**

**Alcohol Misuse (Alc):** Signs of excessive and likely problematic alcohol use.

**Drug Misuse (Drg):** Signs of excessive and likely problematic drug use.

**Impulsivity (Imp):** Behavioral disinhibition resulting in adverse life outcomes.

**Antisocial Behavior (Ant):** Non-conforming and rule-violating behavior and interpersonal exploitation.

**Anger/Aggression (Ang):** Uncontrolled anger and physical aggression.

**Thought Disorder Markers:**

**Psychosis (Pys):** First-rank symptoms of psychosis, auditory and visual hallucinations, plus signs of formal thought disorders

**Paranoid Ideation (Par):** Ideas of persecution, lack of trust, and general suspiciousness.

**Manic Activity (Man):** Mental and physical acceleration and uncontrolled behavior associated with a manic/hypomanic episode.

**Grandiose Ideation (Grd):** Exaggerated sense of personal power, talent, and ability (this scale shades into narcissism).

**Supplemental Scales**

In addition to the core hierarchical dimensions and markers, consider including several additional constructs relevant to psychiatric/mental health treatment. These scales cover important syndromes, attitudes, developmental qualities, and test-taking behaviors that will round out and enhance the assessment.

**Supplemental Constructs to be Considered**

**Eating Concerns**: How much does thinking about food or eating impact one's life?

**Bodily Distress:** Modeled on the new ICD-11 syndrome, it captures symptoms of Gastrointestinal arousal, Musculoskeletal tension, and general symptoms.

**Personality Dysfunction:** Designed to capture identity and self-direction, and reflects the degree or level of personality structure/organization. High scores signal a likely personality disorder.

**Cognitive Complaints:** Captures everyday subjective cognitive experiences of memory, attention, and organizational weakness. It is often associated with internalizing disorders and is typically independent of actual neurocognitive deficits (weak neuropsychological profile).

**Suicidal Ideation:** presence and intensity of suicidal thinking.

**Treatment Attitude:** Attitude toward mental health care (psychotherapy and medications) and expectations.

**Resilience:** A combination of resilience, well-being, and purpose.

**Validity:** Low-frequency items (seldom endorsed) to measure engagement, reading, and accurate responding.

**Supplementary Table S1. Item development process: MTIP scales, construct definitions, and materials consulted.**

| **MTIP Scales** | **Construct Definition** | **Materials Consulted** |
| --- | --- | --- |
| **Validity** |  |  |
| Infrequency | Careless, random, or inconsistent responding | IPIP, INF |
| **Clinical Scales** | DSM Disorders and symptoms |  |
| Depression | Depressive affect and ideation | DSM-5 criteria, HANDS, PHQ-9, DASS |
| Anxiety | Worry, panic, and fear of losing control | DSM-5 criteria, Penn Worry Scale, GAD-7, DASS |
| Social Anxiety | Social avoidance and social inadequacy | DSM-5 criteria, DSM-5 Severity Measure |
| Traumatic Stress | Intrusive memories, avoidant behavior, and physical reactivity to a stressful event | DSM-5 criterion B, PCL-5, DSM-5 Severity Measure |
| Alcohol Misuse | Social, personal, and occupational problems related to alcohol use | DSM-5 criteria, AUDIT |
| Drug Misuse | Social, personal, and occupational problems related to drug use | DSM-5 criteria, DAST |
| Anger/Aggression | Poorly controlled explosive anger and violence | STAB Physical Aggression Scale, BPAQ physical aggression subscale |
| Impulsivity | Behavioral dysregulation and lack of planning | Impulsivity subscale PID-5, BIS/BAS |
| Antisocial Behavior | Disregard for social, ethical, or legal convention | DSM-5 criteria, PID-5 |
| Psychosis | Visual and auditory misperceptions, thought impairment | DSM-5 criteria, Unusual beliefs subscale PID-5 |
| Paranoid Ideation | Ideas of reference and persecution | DSM-5 criteria, PID-5 Suspiciousness subscale |
| Manic Activation | Mental and physical acceleration | DSM-5 criteria, DSM-5 Severity Measure, YMS |
| Grandiose Ideation | Elevated sense of importance, talent, and destiny | DSM-5 criteria for Narcissistic PD, DSM-5 description of Grandiose Delusions, PID-5 Grandiosity subscale |
| **Transdiagnostic Scales** | Core dimensions of psychopathology |  |
| **Supplemental Scales** |  |  |
| Cognitive Complaints | Memory, attention, and organization complaints | CPFQ, PDQ |
| Body Distress | Vague nonspecific physical symptoms | DSM-5 Criteria, SSS-8 |
| Eating Issues | Preoccupation and worries about food | DSM-5 Criteria, EAT |
| Resilience | Psychological well-being and agency | SOS-10, DASS, RSES |
| Personality Dysfunction | Instability of self and other representations | DSM-5 criterion A, SCORS-G |
| Suicidal Ideation | Preoccupation with thoughts of death and dying | SBQ-R |
| Treatment Attitude | Attitude toward mental health treatment | CQ |

Note. Materials Consulted

**AUDIT:** Saunders, J.B., Aasland, O.G., Babor, T.F., de la Fuente, J.R., & Grant, M. (1993).

Development of the WHO collaborative project on early detection of persons with harmful

alcohol consumption. *Addiction, 88,* 791-804.

**BIS/BAS:** Carver, C. S., & White, T. L. (1994). Behavioral inhibition, behavioral activation, and

affective responses to impending reward and punishment: The BIS/BAS scales. *Journal*

*of Personality and Social Psychology,* 67, 319–333.

**CPFQ:** Fava, M., Iosifescu, D. V., Pedrelli, P., and Baer, L. (2009). Reliability and validity of the Massachusetts General Hospital cognitive and physical functioning questionnaire, *Psychotherapy and* *Psychosomatics, 78,* 91-97.

**CQ:** Miller WR, Johnson WR. A natural language screening measure for motivation to change.

(2008). Addictive Behaviors. 33(9):1177–1182.

**DASS:** Sinclair SJ, Siefert CJ, Slavin-Mulford JM, Stein MB, Renna M, **Blais MA**. (2012).

Psychometric evaluation and normative data for the depression, anxiety, and stress scales-

21 (DASS-21) in a nonclinical sample of U.S. adults. *Evaluation and the Health*

*Professions.* 2012;35(3):259-279.

**DAST:** Skinner, H. A. (1983). The drug abuse screening test. *Journal of Addictive Behaviors, 7*,

363-371.

**DSM criteria**: DSM-5 diagnostic criteria (APA. (2013). *Diagnostic and statistical manual of*

*mental disorders: DSM-5* (5 ed.). Washington, DC: American Psychiatric Publishing

Incorporated.

**DSM-5 Severity Measures**: Section III, Emerging Measures and Models of DSM-5, APA.

(2013). *Diagnostic and statistical manual of mental disorders: DSM-5* (5 ed.).

Washington, DC: American Psychiatric Publishing Incorporated

**GAD-7:** Spitzer RL, Kroenke K, Williams JBW, Löwe B. (2006). A Brief Measure for Assessing

Generalized Anxiety Disorder: The GAD-7. Archives of Internal Medicine. 166(10):1092–

1097. doi:10.1001/archinte.166.10.1092

**HANDS:** Baer L, Jacobs DG, Meszler-Reizes J, Blais MA, Fava M, Kessler R, Magruder K,

Murphy J, Kopans B, Cukor P, Leahy L, O'Laughlen J. (2000). Development of a brief

Screening instrument: The HANDS. *Psychotherapy and Psychosomatics*; 69(1):35-41.

**INF:** Siefert CJ, Stein M, Sinclair SJ, Antonius D, Shiva A, **Blais MA**. (2012). Development and initial

validation of a scale for detecting inconsistent responding on the personality

assessment inventory-short form. *Journal of Personality Assessment.* 94(6):601-606.

**IPIP:** Goldberg, L. G., Johnson, J. H., Eber, H. W., Hogan, R., Ashton, M. C., Cloninger, R.,

Gough, H. G. (2006). International Item Pool and future public-domain personality

measures, *Journal of Research in Personality*, 40, 84–96 (ipip.ori.org)

**PCL-5:** Lang, A J., Wilkins, K., Roy-Byrne, P. P., Golinelli, D., … Stein, M. B. (2012). Abbreviated PTSD checklist (CL) as a guide to clinical response. General Hospital Psychiatry, 34; 332 – 338.

**PCP:** Sullivan, J. L., Edgley, K., & Dehoux, E. (1990). A survey of multiple sclerosis: Part 1: Perceived cognitive problems and compensatory strategy use. *Canadian Journal of Rehabilitation, 4,* 99–105.

**PID-5:** Krueger, R.F., Derringer, J., Markon, K.E., Watson, D., & Skodol, A.E. (2012). Initial construction of a maladaptive personality trait model and inventory for DSM-5. *Psychological Medicine, 42*, 1879-1890.

**PHQ-9:** Kroenke K, Spitzer RL, Williams JB. (2001). The PHQ-9: validity of a brief depression

severity measure. *Journal of General Internal Medicine, 16*, 606-613.

**PSWQ:** Meyer TJ, Miller ML, Metzger RL, Borkovec TD. (1990). Development and validation

of the Penn State Worry Questionnaire. Behavioral Research and Therapy, 28(6), 487-95.

doi: 10.1016/0005-7967(90)90135-6. PMID: 2076086.

**RSES:** Sinclair SJ, Blais MA, Gansler DA, Sandberg E, Bistis K, LoCicero A. (2010).

Psychometric properties of the Rosenberg Self-Esteem Scale: Overall and across

demographic groups living within the United States. *Evaluation and Healthcare*

*Professions.* 33(1):56-80.

**SBQ-R:** Osman, A., Bagge, C. L., Gutierrez, P. M., Konick, L. C., Kopper, B. A., & Barrios, F.

X. (2001). The Suicidal Behaviors Questionnaire-Revised (SBQ-R): Validation with

clinical and nonclinical samples. *Assessment, 8*, 443-454.

**SCORS-G:** Stein MB, Slavin-Mulford J, Sinclair SJ, Siefert CJ, **Blais MA**. (2012). Exploring the

construct validity of the Social Cognition and Object Relations Scale in a clinical sample.

*Journal of Personality Assessment.* 94(5):533-540.

**SOS-10:** Blais MA, Lenderking WR, Baer L, deLorell A, Peets K, Leahy L, Burns C. (1999).

Development and initial validation of a brief mental health outcome measure. *Journal*

*Of Personality Assessment*;73(3):359-373.

**SSS-8:** Gierk B, Kohlmann S, Kroenke K, Spangenberg L, Zenger M, Brähler E, et al. The

Somatic Symptom Scale-8 (SSS-8): a brief measure of somatic symptom burden. Journal

of the American Medical Association Internal Medicine 2014;174:399–407.

https://doi.org/10.1001/ jamainternmed.2013.12179.

**STAB:** Burt, S. A. & Donnellan, M. B. (2009). Development and validation of the Subtypes of

Antisocial Behavior Questionnaire. *Aggressive Behavior, 35*, 376-398.

**Zung Depression Scale**: Zung, W. W. (1965). Self-rated depression scale. *Archives of General*

*Psychiatry*, 12, 63 – 70.

**Supplementary Table S2. Estimates of Variance in MTIP Clinical and Transdiagnostic Scale Scores Attributable to Demographic Variables**

|  |  | **% of variance** |  |  |  |
| --- | --- | --- | --- | --- | --- |
| **MTIP Scales** | **Age** | **Sex** | **Race** | **Ed** | **Total Variance** |
| Dep | 4.2 | 0.01 | 0.1 | 2.0 | 6.31 |
| Anx | 5.9 | 3.2 | 0.4 | 1.4 | 10.90 |
| SocA | 4.2 | 1.2 | 0.07 | 1.1 | 6.57 |
| Ts | 2.1 | 1.5 | 0.2 | 0.9 | 4.70 |
| Alc | 0.6 | 0.8 | 0.3 | 0.2 | 1.90 |
| Drg | 0.9 | 0.6 | 0.2 | 0.6 | 2.30 |
| Ang | 1.1 | 0.1 | 1.8 | 0.8 | 3.80 |
| Imp | 1.8 | 3.2 | 1.5 | 0.2 | 6.70 |
| Ant | 2.9 | 9.4 | 0.3 | 0.6 | 13.20 |
| Psy | 1.7 | 0.3 | 1.3 | 0.7 | 4.00 |
| Par | 1.4 | 0.05 | 1.8 | 1.0 | 4.25 |
| Man | 4.5 | 0.01 | 0.3 | 0.2 | 5.01 |
| Grd | 1.0 | 0.5 | 0.5 | 0.7 | 2.70 |
| TED | 6.3 | 1.7 | 0.4 | 2.2 | 10.60 |
| TBD | 2.9 | 5.3 | 0.6 | 0.3 | 9.10 |
| TTD | 3.5 | 0.4 | 1.8 | 0.1 | 5.80 |
| GPS | 7.7 | 0.01 | 0.6 | 1.6 | 9.91 |

N=640. MTIP Scales: Dep= Depression; Anx= Anxiety; Ts= Traumatic Stress; SocA= Social Anxiety; Alc= Alcohol Misuse; Drg= Drug Misuse; Ang= Anger/Aggression; Imp = Impulsivity; Ant= Antisocial Behavior; Psy= Psychosis; Par= Paranoid Ideation: Man= Manic Activation; & Grd= Grandiose Ideation. TED = Transdiagnostic Emotion Dysregulation; TBD = Transdiagnostic Behavior Dysregulation; TTD = Transdiagnostic Thought Dysregulation; GPS = Global Psychopathology Severity; % of variance = percent of variance accounted for by the individual demographic variables; Total Variance = the total variance in the MTIP scale accounted for by all demographic variables.

**Supplementary Table S3. Raw Score Means and Effect Size Differences by Sex (Males, n = 295, & Females, n = 342).**

| **MTIP Scales** | **Male** | **Female** | **P Value** | **Effect**  **Size*** | **ES 95% CI** |
| --- | --- | --- | --- | --- | --- |
| Dep | 12.01 | 11.58 | 0.33 | 0.07 | -0.07 – 0.23 |
| Anx | 12.03 | 13.45 | 0.003 | 0.23 | 0.07 – 0.39 |
| SocA | 8.58 | 9.16 | 0.09 | 0.13 | 0.25 – 0.28 |
| Ts | 8.12 | 8.68 | 0.04 | 0.16 | 0.01 – 0.31 |
| Alc | 5.04 | 4.47 | < 0.001 | 0.28 | 0.12 – 0.43 |
| Drg | 4.67 | 4.25 | 0.002 | 0.25 | 0.09 - 0.40 |
| Ang | 5.63 | 5.30 | 0.08 | 0.13 | -0.01 – 0.29 |
| Imp | 7.99 | 6.80 | <0.001 | 0.42 | 0.27 – 0.58 |
| Ant | 9.09 | 7.33 | <0.001 | 0.64 | 0.48 – 0.80 |
| Psy | 6.20 | 5.79 | 0.01 | 0.20 | 0.05 – 0.36 |
| Par | 5.98 | 5.69 | 0.17 | 0.11 | -0.04 – 0.26 |
| Man | 6.05 | 5.83 | 0.26 | 0.09 | -0.06 – 0.24 |
| Grd | 8.53 | 7.61 | <0.001 | 0.27 | 0.13 – 0.41 |
| **Transdiagnostic** |  |  |  |  |  |
| TED | 40.78 | 42.89 | 0.09 | 0.01 | -0.28 – 0.025 |
| TBD | 32.43 | 28.18 | <0.001 | 0.51 | 0.35 – 0.67 |
| TTD | 26.75 | 25.27 | 0.01 | 0.19 | 0.04 – 0.35 |
| GPS | 99.97 | 96.35 | 0.07 | 0.14 | -0.01 – 0.29 |

* Effect Size (ES) = Cohen’s d. Dep= Depression; Anx= Anxiety; Ts= Traumatic Stress; SocA= Social Anxiety; Alc= Alcohol Abuse; Drg= Drug Abuse; Ang= Anger/Aggression; Imp = Impulsivity; Ant= Antisocial Behavior; Psy= Psychosis; Par= Paranoid Ideation: Man= Manic Activation; & Grd= Grandiose Ideation. TED = Transdiagnostic Emotion Dysregulation; TBD = Transdiagnostic Behavior Dysregulation; TTD = Transdiagnostic Thought Dysregulation; and GPS = Global Psychopathology Severity.

**Supplementary Table S4. Final Regression Model for the DSM-XC prediction of the MTIP Global Psychopathology Severity Scale (GPS).**

| **Predictor**  ***DSM-XC Scales*** | **R** | **Adjusted**  **R^2^** | **Std. Error** | ***R^2^ ∆*** | ***F change*** | ***p*** |
| --- | --- | --- | --- | --- | --- | --- |
| XC-ANX | 0.747 | 0.557 | 7.1553 | 0.558 | 804.787 | <0.001 |
| XC-PSY | 0.814 | 0.661 | 6.2567 | 0.105 | 197.429 | <0.001 |
| XC-DEP | 0.850 | 0.722 | 5.6686 | 0.061 | 140.014 | <0.001 |
| XC-MAN | 0.868 | 0.751 | 5.3631 | 0.029 | 75.539 | <0.001 |
| XC-ANG | 0.873 | 0.760 | 5.2641 | 0.009 | 25.091 | <0.001 |
| XC-COG | 0.877 | 0.767 | 5.1881 | 0.007 | 19.722 | <0.001 |
| XC-SUD | 0.881 | 0.773 | 5.1180 | 0.007 | 18.450 | <0.001 |
| XC-PF | 0.882 | 0.776 | 5.0896 | 0.003 | 8.077 | 0.005 |
| XC-OCD | 0.883 | 0.777 | 5.0765 | 0.001 | 4.272 | 0.039 |
|  |  |  |  |  |  |  |

Note. N=640. GPS = MTIP Global Psychopathology Severity Scale; DSM-XC = DSM-5 Cross-Cutting Measure; DSM-XC scale abbreviations are prefixed XC-: XC-DEP = Depression; XC-ANX = Anxiety; XC-SOM = Somatization; XC-SUD = Substance Use Disorder; XC-ANG = Anger; XC-COG = Cognitive Concerns; XC-PSY = Psychosis; XC-MAN = Manic Activation; XC-PF = Personality Functioning; Cog= Cognitive Concerns; & OCD = Obsessive-Compulsive Disorder.

**Supplementary Table S5. Final Regression Models for the DSM-XC prediction of the MTIP Transdiagnostic Scales: TED, TBD, and TTD.**

| **Predictor**  ***DSM-XC Scales*** | **R** | **Adjusted**  **R^2^** | **Std. Error** | ***R^2^ ∆*** | ***F change*** | ***p*** |
| --- | --- | --- | --- | --- | --- | --- |
| *MTIP TED Scale* | | | | | | |
| XC-ANX | 0.858 | 0.736 | 2.0655 | 0.737 | 1786.038 | <0.001 |
| XC-DEP | 0.905 | 0.819 | 1.7111 | 0.083 | 292.713 | <0.001 |
| XC-COG | 0.908 | 0.825 | 1.6852 | 0.006 | 20.666 | <0.001 |
| XC-SOM | 0.910 | 0.827 | 1.6737 | 0.003 | 9.838 | 0.002 |
| XC-PF | 0.911 | 0.828 | 1.6661 | 0.002 | 6.778 | 0.009 |
| XC-MAN | 0.912 | 0.831 | 1.6582 | 0.002 | 8.582 | 0.004 |
| *MTIP TBD Scale* | | | | | | |
| XC-OCD | 0.554 | 0.294 | 9.5060 | 0.295 | 267.501 | <0.001 |
| XC-SUD | 0.649 | 0.419 | 8.6270 | 0.120 | 137.641 | <0.001 |
| XC-ANG | 0.689 | 0.472 | 8.2208 | 0.054 | 65.510 | <0.001 |
| XC-MAN | 0.715 | 0.508 | 7.9405 | 0.036 | 46.683 | <0.001 |
| XC-PSY | 0.729 | 0.528 | 7.7728 | 0.021 | 28.705 | <0.001 |
| XC-SOM | 0.733 | 0.533 | 7.7354 | 0.005 | 7.144 | 0.008 |
| XC-COG | 0.736 | 0.537 | 7.7024 | 0.005 | 3.442 | 0.011 |
| XC-ANX | 0.739 | 0.540 | 7.6769 | 0.004 | 5.196 | 0.023 |
| XC-SUI | 0.741 | 0.542 | 7.6584 | 0.003 | 4.057 | 0.044 |
| *MTIP TTD Scale* | | | | | | |
| XC-PSY | 0.635 | 0.402 | 8.6318 | 0.403 | 430.217 | <0.001 |
| XC-MAN | 0.709 | 0.501 | 7.8875 | 0.099 | 127.096 | <0.001 |

Note. N=640. MTIP TED = Transdiagnostic Emotion Dysregulation; TBD = Transdiagnostic Behavior Dysregulation; TTD = Transdiagnostic Thought Dysregulation; DSM-XC = DSM-5 Cross-Cutting Measure; DSM-XC scale abbreviations are prefixed XC-: XC-DEP = Depression; XC-ANX = Anxiety; XC-SOM = Somatization; XC-SUD = substance use disorder; XC-ANG = Anger; XC-COG = Cognitive Concerns; XC-PSY = Psychosis; XC-MAN = Manic Activation;
